# A Type I IFN-Inducing Oncolytic Virus Improves NK Cell-Mediated Killing of Tumor Cells In Vitro Through Multiple Mechanisms

**DOI:** 10.3390/v17070897

**Published:** 2025-06-25

**Authors:** Elisabeth M. Shiffer, Jeremiah L. Oyer, Alicja J. Copik, Griffith D. Parks

**Affiliations:** Burnett School of Biomedical Sciences, College of Medicine, University of Central Florida, Orlando, FL 32827, USA; elisabeth.shiffer@ucf.edu (E.M.S.); jeremiah.oyer@ucf.edu (J.L.O.); alicja.copik@ucf.edu (A.J.C.)

**Keywords:** natural killer cells, parainfluenza virus 5, oncolytic virus, type 1 interferon, immunotherapeutic

## Abstract

Natural killer (NK) cell adoptive immunotherapy is a promising therapeutic approach in which NK cells perform targeted lysis of tumor cells. Oncolytic viruses are also effective cancer therapeutic agents due to their ability to selectively target and kill tumor cells. Combination therapies that integrate NK cells and oncolytic viruses have been shown to enhance tumor killing compared to individual treatment strategies alone. Using in vitro expanded human NK cells (PM21-NK cells), we tested the relative ability of tumor cells infected with WT parainfluenza virus 5 (PIV5), which is a poor inducer of type 1 interferon (IFN-I), versus PIV5 P/V gene mutant, which is a strong inducer of IFN-I synthesis, to modulate NK cell activities. Both WT and P/V mutant viruses were capable of infecting PM21-NK cells and caused extensive cytopathic effects. Co-culturing of PM21-NK cells with virus-infected tumor cells resulted in spread of WT PIV5 to naïve NK cells, but NK cells were protected from spread of the P/V mutant virus by IFN-I induction. Direct treatment of PM21-NK cells with IFN-I or media from P/V-virus-infected tumor cells enhanced NK cell cytotoxicity, at least in part due to upregulation of the death ligand, TRAIL. IFN-I-treated PM21-NK cells also showed a decrease in IFN-γ secretion, a cytokine we have previously shown to reduce PM21-NK cell tumor killing. Our results highlight multiple mechanisms by which an IFN-I-inducing oncolytic virus can enhance NK-cell-mediated killing of target virus-infected and uninfected tumor cells.

## 1. Introduction

Natural killer (NK) cells are powerful effector lymphocytes of the innate immune system that function in targeted lysis of tumor and virus-infected cells [1]. Once activated by recognition of a target cell, NK cells can release cytotoxic granules containing perforin and granzyme, causing lysis of the target cell [2]. NK cells can also perform death-ligand-induced apoptosis of target cells by interaction of death ligands such as TNF-related apoptosis-inducing ligands (TRAIL) with their respective receptors, TRAIL-R1/-R2, on the surface of target cells [3,4]. NK cell adoptive immunotherapy is a novel cancer therapeutic approach being developed in which allogenic NK cells function as potent anti-cancer agents [5,6,7]. Advances in in vitro NK cell generation methods have expanded the potential of using NK cells for adoptive immunotherapeutic applications. We have developed a particle-based ex vivo expansion method in which NK cells are stimulated with PM21 particles, derived from the plasma membrane of cells engineered to express NK-cell-stimulating ligands 41-BBL and membrane bound IL-21 [8,9]. PM21-particle stimulation of PBMCs yields > 1000-fold expansion of PM21-NK cells that exhibit heightened cytotoxicity towards tumor cells [10,11].

There is increasing interest in developing anti-cancer immunotherapeutic agents based on oncolytic viruses: naturally occurring and genetically engineered viruses that selectively target and lyse tumor cells [12]. In addition to killing tumor cells, oncolytic viruses can induce anti-tumor immune responses that aid in the clearance of tumors. A key attribute of an oncolytic virus is the ability to selectively infect and kill tumor cells while exhibiting minimal pathogenicity to healthy tissues, referred to as oncotropism [13,14]. One way to achieve oncotropism is by using an interferon (IFN)-sensitive virus that is unable to replicate in healthy cells but that propagates in tumor cells with defective IFN pathways [15,16].

Paramyxoviruses are gaining attention as oncolytic vectors partly due to their ability to modulate anti-tumor immune responses [17,18,19]. Wild-type parainfluenza virus 5 (WT PIV5) is not suitable as an oncolytic virus candidate due to the role of the V protein in blocking antiviral responses and cytopathic effect within infected cells [20,21,22]. We have previously described a PIV5 mutant with mutations in the P/V gene that render the V protein defective in blocking IFN-I signaling and synthesis. These changes convert WT PIV5 into a highly cytopathic and tumor-restricted oncolytic virus [22,23,24,25,26], that is also a potent inducer of cytokines, including type I interferon (IFN-I). Here, we use WT PIV5 and the P/V mutant to dissect the roles of tumor cell-derived IFN-I in altering the efficacy of NK cell and oncolytic virus combination therapy.

Combination therapies that integrate NK cells with oncolytic viruses offer unique mechanisms to improve targeting and killing of tumor cells compared to individual treatment strategies alone. Oncolytic-virus-infected tumor cells express surface viral glycoproteins [27,28] and altered levels of cellular proteins and cytokines [29,30], both of which have been shown to enhance NK-cell-mediated killing of tumor cells. These properties, including the natural cytotoxicity of NK cells towards virus-infected cells, highlight the potential for enhanced efficacy of oncolytic virus and NK cell combination anti-cancer therapeutic approaches. We have previously reported that infection of lung tumor cells in vitro with the P/V mutant enhances their killing by PM21-NK cells [28]. Here we show that the induction of IFN-I by P/V-virus-infected tumor cells can potentiate PM21-NK cell anti-tumor killing through multiple mechanisms.

Despite research supporting the use of oncolytic virus and NK cell combination therapy, there is limited understanding of the rates and implications of off-target oncolytic virus infection of NK cells. Oncolytic viruses replicate and release progeny virions from infected tumor cells that can subsequently infect neighboring cells, including NK cells. Due to the cytopathic nature of oncolytic viruses, blocking infection of NK cells during combination therapy is important for maintaining sufficient quantities of healthy NK cells for efficient tumor killing. IFN-I is a key cytokine produced during P/V mutant oncolytic virus infection and is known to induce an antiviral state that protects neighboring cells from virus infection [31,32]. Here we show that IFN-I secreted from P/V-mutant-infected tumor cells contributes to enhanced killing by PM21-NK cells through three mechanisms. First, IFN-I protects PM21-NK cells from the cytopathic effects of oncolytic virus infection. Secondly, IFN-I directly enhances the cytotoxic potency of PM21-NK cells towards lung, neuroblastoma, and rhabdomyosarcoma tumor cells. Finally, we show that IFN-I-treated PM21-NK cells have decreased production of IFN-γ, a cytokine with dual effects on inhibiting tumor grow but also influencing tumor survival. We and others have previously shown that IFN-γ also upregulates expression of NK cell inhibitory ligands on the surface of target tumor cells and decreases NK cell killing [33]. Together, these results highlight the importance of using an IFN-I inducing oncolytic virus during NK cell and oncolytic virus combination therapy.

## 2. Materials and Methods

### 2.1. Cells Lines, Viruses, and Infections

Cultures of A549, SK-N-AS, and RD (ATCC) were grown at 37 °C under a humidified 5% CO_2_ atmosphere in Dulbecco modified Eagle medium (DMEM, Gibco, Thermo Fisher Scientific, Waltham, MA, USA) supplemented with 10% heat-inactivated fetal bovine serum (HI FBS, Gibco, Thermo Fisher Scientific). Transduction of A549, SK-N-AS, and RD cells using NucLight Red lentivirus (Sartorius, Göttingen, Germany) followed by 1 μg/mL puromycin selection was used to generate cells expressing a nuclear red fluorescence protein (NLR cells).

Parainfluenza virus 5 (WT PIV5) and P/V-mutant expressing green fluorescence protein (GFP) were grown in Vero cells and titered on CV-1 cells as previously described [24]. A549 cells were infected with virus diluted in DMEM with 10% bovine serum albumin (BSA, Gibco, Thermo Fisher Scientific) for 1 h or mock-infected with media alone. Following incubation, A549 cells were washed with phosphate-buffered saline (PBS) and incubated with DMEM supplemented with 2% HI FBS. NK cells were infected with virus diluted in Roswell Park Memorial Institute medium (RPMI, Gibco, Thermo Fisher Scientific) supplemented with 10% HI FBS for 1 h. Following incubation, NK cells were centrifuged and incubated in fresh RPMI with 10% HI FBS and 100 U/mL interlueukin-2 (IL2, PeproTech, Cranbury, NJ, USA).

### 2.2. PM21-NK Cell Preparation and Cryopreservation

PM21 particles were generated, and NK cells were expanded from peripheral blood mononuclear cells (PBMCs) as previously described [8]. PBMCs were depleted of T cells (EasySep CD3 positive selection kit; STEMCELL Technologies, Cambridge, MA, USA) and then cultured with 100 U/mL interlueukin-2 (IL2, PeproTech) and 200 µg/mL PM21 particles in SCGM media (Cell Genix, Sartorius, Freiburg, Germany) supplemented with 10% non-HI FBS for 7 days. NK cell cultures post day 7 were maintained in RPMI supplemented with 10% non-HI FBS and 100 U/mL IL-2. NK cells were cryopreserved at a concentration of 1 × 10^7^ cells/mL in a solution of 50% RPMI, 40% FBS, and 10% dimethyl sulfoxide (DMSO, Sigma-Aldrich, St. Louis, MO, USA) as previously described [10].

### 2.3. Flow Cytometry

Twenty-four-well plates of NK cells were treated as described in the figure legends. NK cells were identified by staining with PE-conjugated CD56 antibody (catalog #362508, BioLegend, San Diego, CA, USA). Infected NK cells were identified by flow cytometry as CD56-positive and expressing virus-derived green fluorescent protein (GFP). Cell surface expression of PIV5 F and HN glycoproteins on infected NK cells were quantified using a mouse anti-PIV5 polyclonal antiserum ([34]) followed by secondary staining using anti-mouse AlexaFluor 405 (catalog #A31553, Thermo Fisher Scientific, Waltham, MA, USA). Surface expression of TRAIL on NK cells was quantified using conjugated TRAIL antibody (catalog #308210, Biolegend). The flow cytometry gating strategy for TRAIL staining of NK cells is shown in Supplemental Figure S1. Untreated A549 cells were stained with conjugated TRAIL-R1 and TRAIL-R2 antibodies (catalog #307207 and #307207, Biolegend). For all flow cytometry assays, cells were analyzed using the CytoFLEX (Beckman Coulter, Brea, CA, USA) and CytExpert software (Beckman Coulter, version 2.4) and FlowJo software (BD Life Sciences, version 10.6.2) was used to analyze 10,000 independent events.

Cytopathic effect (CPE) of virus infection was determined by analyzing NK cells for negative propidium iodide (PI, BD Bioscience, Franklin Lakes, NJ, USA) staining as described by the manufacturer. Cell counts were obtained by normalizing cytometer events by the run time for each sample and multiplying by the flow rate. The flow cytometry gating strategy for NK cells counts and PI negative staining are shown in Supplemental Figure S2. For IFN-I blocking assays, media from A549 infections were UV-inactivated, as described below and treated with 1:50 dilution of IFN-I neutralizing antibody mixture, directed against several type I IFNs (catalog #39000, PBL Assay Science, Piscataway, NJ, USA) for 30 min before using the media to treat NK cells.

### 2.4. Western Blotting

Six-well plates of cells, populated at 2 × 10^5^ NK cells/mL, were treated as described in the figure legends. Cells were lysed using protein lysis buffer, resolved on 12% sodium dodecyl sulfate/polyacrylamide gel electrophoresis (SDS-PAGE) gels and transferred to nitrocellulose membranes (Bio-Rad, Hercules, CA, USA). Samples were normalized to β-Actin (1:20,000 dilution, catalog #A5316, Sigma-Aldrich, St. Louis, MO, USA) then probed with antibodies for PIV5 NP, P, (1:2000 dilutions, [35]), and STAT-1 (1:2000, catalog #9172, Cell Signaling Technology, Danvers, MA, USA). Blots were visualized using anti-mouse horseradish peroxidase (HRP)-conjugated antibodies (Sigma-Aldrich) and chemiluminescence (Thermo Fisher Scientific, Waltham, MA, USA).

### 2.5. Reverse Transcription and Real-Time PCR

Ten cm dishes populated at 1 × 10^6^ NK cells/mL were treated as described in the figure legends. RNA extraction was performed using TRIzol (Invitrogen, Thermo Fisher Scientific, Waltham, MA, USA) as described by the manufacturer. Briefly, 1 µg of total RNA was used to generate cDNA using Oligo d(T)_16_ and TaqMan Reverse Transcription Reagents (Applied Biosystems, Foster City, CA, USA). Bio-Rad CFX Connect Real-Time and Fast SYBR™ Green Master Mix (Applied Biosystems) was used to perform quantitative real-time PCR. Relative gene expression was determined using CFX Manager Software, version 2.3 (Bio-Rad, Hercules, CA, USA). Primer sequences for OAS2, IFIT, and IFITM1 genes were as described [36]. Primer sequences for viral proteins F, HN, M and NP genes were as described [37].

### 2.6. ELISA

Cells were treated as described in the figure legends; media were collected and analyzed using ELISA kits for Human IFN-β (catalog #41410-2, PBL Assay Science, Piscataway, NJ, USA) or IFN-γ (catalog #550612, BD Biosciences, Franklin Lakes, NJ, USA) as described by the manufacturers.

### 2.7. UV Media Inactivation and Cytokine Treatment

A549 cells were infected at an MOI of 10, media were collected at 24 hpi and UV-inactivated for 15 min before treating NK cells as described in the figure legends. For cytokine assays, NK cells were treated with 1000 Units/mL of universal interferon type 1 (IFN-1, catalog #11200-2, PBL Assay Science, Piscataway, NJ, USA), unless otherwise noted, and incubated for 18 h. As described in the figure legends, NK cells were stimulated with an activating cocktail of PMA and Ionomycin (catalog #423302, BioLegend, San Diego, CA, USA) as described by the manufacturer.

### 2.8. Cell Viability and Cytotoxicity Assays

Real-time cell viability assays were performed using an IncuCyte instrument (Sartorius, Gottingen, Germany) as previously described [28]. Briefly, tumor cells expressing red fluorescence protein (NLR cells) were plated in 96-well plates (catalog #07200704, Corning, Thermo Fisher Scientific, Waltham, MA, USA) at 7000 cells/well and incubated overnight. PM21-NK cells, treated as described in the figure legends, were added at varying effector to target (E:T) ratios. Plates were incubated at 37 °C under 5% CO_2_ within the IncuCyte system and imaged every 2 h using a 10× objective with red, green, and phase channels. The red object count (ROC) corresponding to tumor cell nuclei was calculated for each well. The values for the ROC at each time point were expressed as a percentage of the value at time zero (ROC^t0^). The percent cytotoxicity was calculated by normalizing the ROC of the wells containing NK cells plus tumor cells to the ROC of the wells containing tumor cells alone in the absence of NK cells.

For antibody blocking experiments, NK cells were incubated with 10 μg/mL purified TRAIL or isotype control antibody (catalog #308202 and #400101, Biolegend, San Diego, CA, USA) at 37 °C for 1 h prior to addition of tumor cells and beginning IncuCyte scanning.

### 2.9. Figures, Statistics, and Images

Values in all panels are the mean of three biological replicates. Statistical analysis was performed using GraphPad (version 10.2.3) 2way ANOVA. In all figures, *** and **** indicate a *p*-value of <0.001 and <0.0001, respectively.

## 3. Results

### 3.1. PM21-NK Cells Are Susceptible to PIV5 Infection

For the experiments below, two different versions of PIV5, both of which express GFP as an additional gene inserted between HN and L, were used for PM21-NK cell and target tumor cell infections: (1) WT PIV5, which is a poor inducer of IFN-I [20] and blocks IFN signaling due to V protein targeting of STAT-1 [38], and (2) a P/V mutant virus, which is a strong inducer of IFN-I and is defective in blocking IFN-I signaling [24].

**Figure 1:**
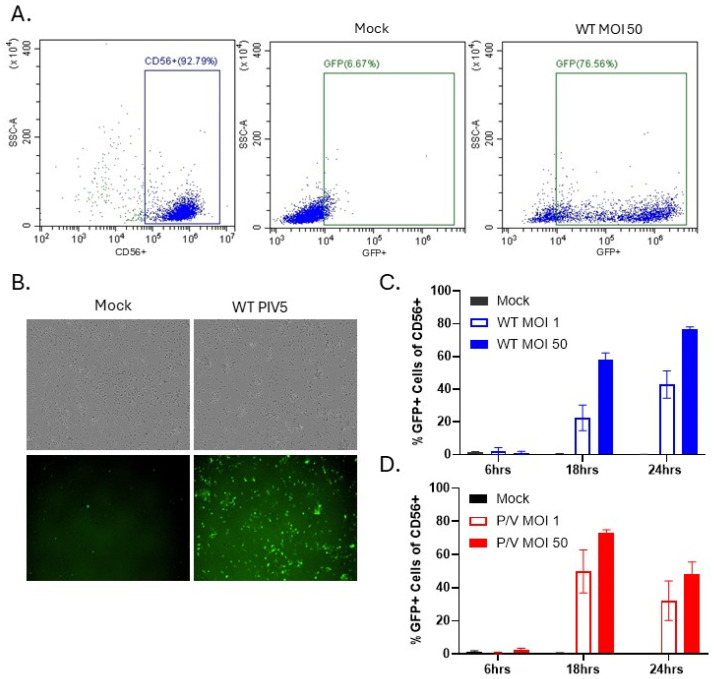
PM21-NK cells are susceptible to PIV5 infection. PM21-NK cells were mock-infected or infected with WT PIV5 or P/V mutant at an MOI of 1 or 50. (**A**,**C**,**D**) At 6, 18, and 24 hpi, cells were stained for CD56 and flow cytometry was used to quantify the percent of GFP-positive CD56+ cells. Representative scatterplots of mock-infected and WT PIV5-infected cells are shown in panel (**A**). Percent of GFP-positive cells following WT PIV5 infection (**C**) and P/V infection (**D**). (**B**) At 24 hpi, cells were examined by brightfield (BF) and fluorescence (FL) microscopy for GFP expression. Values in all panels are the mean of three replicates with error bars representing standard deviation.

To determine the sensitivity of PM21-NK cells to PIV5 infection, NK cells were mock-infected or infected at an MOI of 1 or 50 with WT PIV5 or P/V mutant, and GFP expression was quantified using flow cytometry. Figure 1A shows representative scatter plots of mock-infected and WT PIV5-infected PM21-NK cells, with CD56 staining and GFP expression used to identify infected NK cells in the population. As shown in Figure 1B, brightfield (BF) and fluorescence (FL) microscopy confirmed GFP expression in infected PM21-NK cells at 24 hpi. Quantification of the frequency of GFP-expressing cells by flow cytometry at 6, 18, and 24 hpi showed increasing percentages of GFP-positive cells, with approximately 80% of the cells in the population infected with WT PIV5 by 24 hpi (Figure 1C). GFP expression in P/V-mutant-infected NK cells peaked by 18 hpi, with ~80% GFP-positive cells followed by decreasing GFP expression at 24 hpi (Figure 1D). These data demonstrate that NK cells are susceptible to efficient infection by both WT PIV5 and P/V mutant virus.

**Figure 2:**
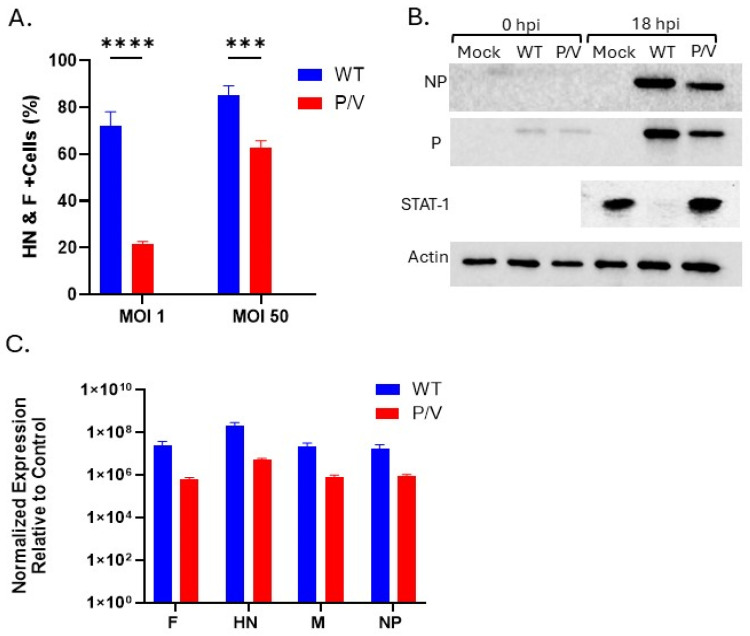
PM21-NK cells are permissive to PIV5 infection. PM21-NK cells were mock-infected or infected with WT PIV5 or P/V-mutant-infected at an MOI of 1 or 50. (**A**) At 24 hpi, cell surface staining was carried out with polyclonal anti-PIV5 antiserum. Percent of positive cells in the population was quantified by flow cytometry. (**B**) Lysates from mock-infected cells or cells infected with WT PIV5 or P/V-mutant-infected NK cells (MOI of 50) were harvested at 0 or 18 hpi and analyzed by western blotting for STAT-1 and viral proteins NP and P. (**C**) Total cellular RNA was isolated from PM21-NK cells that were mock-infected or infected (MOI of 50) with WT PIV5 or P/V mutant, and levels of viral gene expression were determined by qRT-PCR. Data is expressed as gene levels normalized to mock-infected cell RNA, where untreated sample values are all zero. Values in all panels are the mean of three replicates with error bars representing standard deviation. *** and **** indicates a *p*-value of <0.001 and <0.0001, respectively.

Virally infected NK cells should incorporate PIV5 glycoproteins, providing additional evidence of their susceptibility to infection. To confirm that, PM21-NK cells were infected with WT PIV5 or P/V mutant at an MOI of 1 or 50 and viral protein expression was quantified. Surface expression of PIV5 viral glycoproteins, hemagglutinin neuraminidase (HN), and fusion (F) protein was quantified by flow cytometry with a polyclonal antiserum that binds to both proteins. NK cell infection at an MOI of 50 with WT PIV5 resulted in >80% of cells in the population expressing viral glycoproteins on their surface. P/V mutant infection at an MOI of 50 showed >60% positive cells for HN and F surface expression (Figure 2A). As shown in Figure 2B, western blotting of cell lysates from WT PIV5 and P/V-mutant-infected PM21-NK cells at 0 hpi showed limited presence of viral proteins NP and P, representing input viral inoculum, whereas large quantities of these proteins are seen in 18 hpi lysates. PIV5 V protein blocks host-cell antiviral responses by targeting STAT-1 for degradation, and the P/V mutant has a non-functional V protein that is unable to induce STAT-1 degradation [21,24]. STAT-1 degradation was evident in lysates from WT PIV5-infected cells, confirming the synthesis of functional V protein. These results demonstrate that viral proteins are actively expressed by the infected NK cell and are not the result of surface glycoproteins being passively deposited on the surface of the infected cells by fusing virus particles.

Expression of viral genes in infected PM21-NK cells was further confirmed by RT-qPCR of RNA using primers specific for PIV5 F, HN, matrix (M), and nucleocapsid protein (NP). As shown in Figure 2C, there were ~10^8^ and ~10^6^ increases in viral RNA expression seen for WT PIV5 and P/V mutant infections, respectively. Together, these data demonstrate that PM21-NK cells are susceptible to PIV5 infection and that virus protein expression and shuttling of virus glycoproteins to the surface of the cell occurred.

### 3.2. WT PIV5 and P/V Mutant Are Cytopathic to PM21-NK Cells

**Figure 3:**
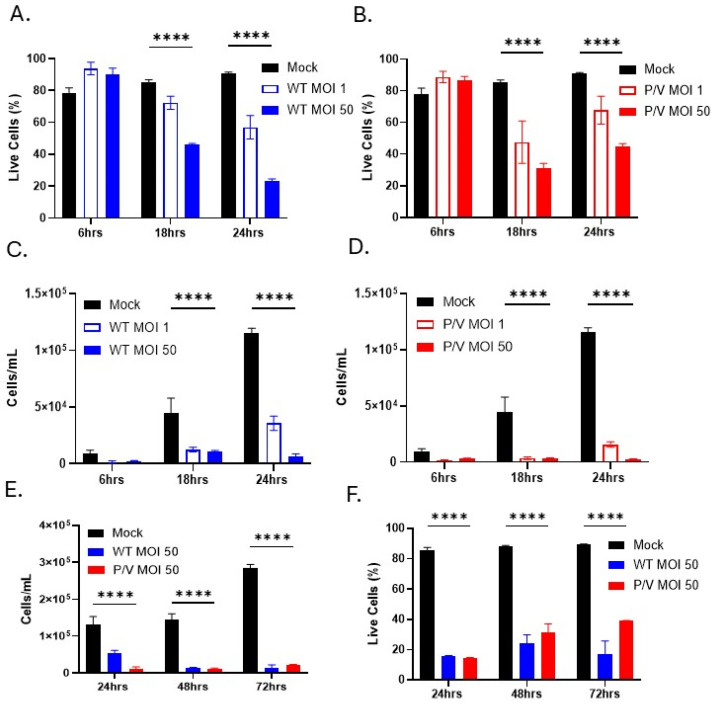
WT PIV5 and P/V mutant are cytopathic to PM21-NK cells. PM21-NK cells were mock-infected or infected at an MOI of 1 or 50 with WT PIV5 or P/V mutant. Cells were harvested at 6, 18, and 24 hpi for analysis by flow cytometry. (**A**,**B**) Infected PM21-NK cells were stained with CD56 antibody and propidium iodide (PI). The percent of CD56+ cells that were PI-negative, characterizing them as live cells, for WT PIV5 infection (**A**) or P/V mutant infection (**B**) was quantified by flow cytometry. (**C**,**D**) Mock-infected or infected PM21-NK cells were stained with CD56 antibody and flow cytometry was used to calculate cell density at 6, 18, and 24 hpi, expressed as cells per milliliter (mL). (**E**,**F**) PM21-NK cells were mock-infected or infected with WT or P/V PIV5 virus (MOI of 50). Cells were harvested at 24, 48, and 72 hpi and flow cytometry was used to calculate cell density (**E**) and percent of PI negative, live cells (**F**) as described above. Values in all panels are the mean of three replicates with error bars representing standard deviation. **** indicates a *p*-value of <0.0001.

Prior work has shown that WT PIV5 is largely non-cytopathic in most non-lymphoid-derived cell lines, whereas the P/V mutant is highly cytopathic [23,24]. To determine the cytopathic effect (CPE) of PIV5 infection of PM21-NK cells, cells were mock-infected or infected with WT PIV5 or P/V mutant at an MOI of 1 or 50. Cell viability was quantified using propidium iodide (PI) staining or viable cell counts by flow cytometry at 6, 18, and 24 hpi. As shown in Figure 3A, PI staining of WT PIV5-infected PM21-NK cells showed that only 20% of cells are PI-negative, classifying them as live cells, at 24 hpi using an MOI of 50 (Figure 3A). Infection with P/V mutant resulted in 40% of cells being PI-negative, live cells at 24 hpi (Figure 3B). While mock-infected cells continued to increase in number due to growth (black bars, Figure 3C,D), cell counts of infected PM21-NK cells show an MOI-dependent reduction in the number of live cells over time (compared to mock-infected cells) following infection with WT PIV5 (Figure 3C) or the P/V mutant (Figure 3D). The time course was extended to determine the number of live cells at 48 and 72 hpi. As shown in Figure 3E,F, PM21-NK cells infected with WT PIV5 and P/V mutant virus showed a significant loss of live cell counts compared to mock-infected cells (Figure 3E), and PI-negative, live cells peak at 20% and 40% for WT and P/V-mutant-infected cells by 72 hpi, respectively (Figure 3F). Together, these data show that infection of PM21-NK cells with either WT or P/V mutant PIV5 results in substantial loss of cell viability.

### 3.3. IFN-I Derived from P/V Mutant Infection of Tumor Cells Can Induce an Antiviral State in PM21-NK Cells and Prevents Tumor-Derived Virus from Spreading to NK Cells

**Figure 4:**
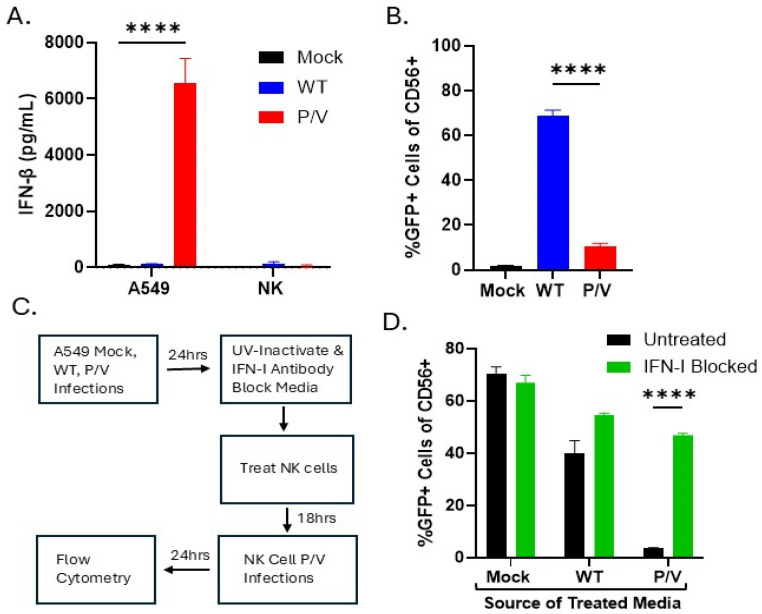
IFN-I production from P/V-mutant-infected tumor cells limits spread of progeny virus to PM21-NK cells. (**A**) A549 or PM21-NK cells were mock-infected or infected with WT or P/V mutant PIV5 at an MOI of 10 or 50, respectively. Media were harvested at 18 hpi and analyzed for levels of IFN-β by ELISA. (**B**) A549 cells were mock-infected or infected with WT PIV5 or the P/V mutant at an MOI of 10. At 24 hpi, PM21-NK cells were co-cultured with infected A549 cells overnight. NK cells were harvested, stained with anti-CD56 antibody, and the percent of GFP-positive NK cells were quantified by flow cytometry. (**C**) The workflow for the experiment in panel D is shown. (**D**) A549 cells were mock-infected or infected at an MOI of 10 with WT PIV5 or P/V mutant. After 24 h, media were collected, UV-treated to inactivate virus, and treated 30 min at 37 °C with an antibody cocktail that neutralizes IFN-I. PM21-NK cells were incubated with treated media for 18 h. Treated PM21-NK cells were then infected with P/V mutant at an MOI of 50. At 24 hpi, cells were stained with CD56 antibody and flow cytometry was performed to quantify the percent of GFP-positive NK cells. Values in all panels are the mean of three replicates with error bars representing standard deviation. **** indicates a *p*-value of <0.0001.

High PIV5 infection rates and CPE in PM21-NK cells are disadvantageous to oncolytic virus and NK cell combination therapy. Given that the P/V mutant is known to be a strong inducer of IFN-I [24], we hypothesized that IFN-I produced by P/V-mutant-infected tumor cells would generate an antiviral state in PM21-NK cells, thus preventing virus infection and NK cell death. As shown in Figure 4A, P/V mutant infection of A549 tumor cells produced very high levels of IFN-beta [24], contrasting with the known poor induction of IFN-beta by WT PIV5 infection [20]. Additionally, as expected PM21-NK cells did not produce detectable IFN-beta following infection with either WT PIV5 of P/V mutant (Figure 4A).

We co-cultured infected A549 target tumor cells with naïve PM21-NK cells to test the ability of progeny PIV5 to spread from tumor cells to NK cells. A549 tumor cells that were mock-infected or infected with WT or P/V mutant PIV5 were co-cultured overnight with naïve PM21-NK cells and the spread of progeny PIV5 to produce GFP-positive NK cells was determined by flow cytometry. As shown in Figure 4B, co-culturing of WT PIV5-infected A549 with PM21-NK cells results in ~70% of NK cells becoming infected, which contrasts with only ~10% of NK cells becoming GFP-positive when co-cultured with P/V-mutant-infected tumor cells.

We tested the hypothesis that it was IFN-I produced by P/V-mutant-infected A549 cells that protected the co-cultured PM21-NK cells from infection. As shown in the diagram in Figure 4C, PM21-NK cells were incubated overnight with UV-inactivated media from infected A549 cells that were left untreated or treated with a cocktail of IFN-I neutralizing antibodies. Media-treated PM21-NK cells were then infected with the P/V mutant as an indicator virus, incubated overnight, then analyzed by flow cytometry for GFP expression. As shown in Figure 4D, PM21-NK cells treated with media from mock-infected A549 cells resulted in 70% GFP expression, indicating they were not protected from PIV5 infection. PM21-NK cells treated with media from WT PIV5-infected tumor cells were efficiently infected with PIV5 and the IFN-I blocking antibodies had little effect. Most importantly, PM21-NK cells treated with media from P/V-mutant-infected A549 cells showed nearly undetectable infection with PIV5, but this infection was increased in the presence of IFN-I blocking antibodies. Together, these data demonstrate that P/V mutant infection of tumor cells causes release of high levels of IFN-I which, in co-cultures with PM21-NK cells, can prevent subsequent infection by progeny virus from the tumor cells.

**Figure 5:**
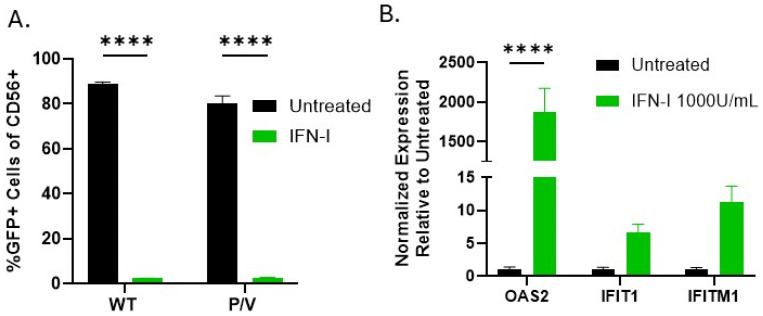
IFN-I induces an antiviral state and prevents PIV5 infection in PM21-NK cells. PM21-NK cells were left untreated or treated with 1000 U/mL IFN-I for 18 h. (**A**) Pretreated PM21-NK cells were infected at an MOI of 50 with WT PIV5 or P/V mutant overnight. Cells were stained with CD56 antibody and flow cytometry was used to quantify the percent of GFP-positive NK cells. (**B**) PM21-NK cells were treated as in panel A above, and total cellular RNA was isolated at 18 hp treatment. Levels of interferon-stimulated genes (ISGs) OAS2, IFIT1, and IFITM1 were determined by qRT-PCR. Data is expressed as gene levels normalized to untreated samples, where untreated sample values are all zero. Values in all panels are the mean of three replicates with error bars representing standard deviation. **** indicates a *p*-value of <0.0001.

To directly confirm that IFN-I can induce an antiviral state in NK cells, PM21-NK cells were left untreated or treated with IFN-I for 18 h. NK cells were then infected with WT PIV5 or P/V mutant and analyzed at 24 hpi by flow cytometry for GFP expression. As shown in Figure 5A, PM21-NK cells that were pretreated with IFN-I demonstrated nearly undetectable levels of virus-derived GFP expression compared to untreated cells, which show >80% infection rates. Interferon-stimulated genes (ISGs) known to be associated with PIV5 infection were analyzed by RT-qPCR following IFN-I treatment of PM21-NK cells. IFN-I treatment showed strong induction of three ISGs: OAS2, IFIT1, and IFITM1 (Figure 5B). Taken together, the protection from PIV5 infection and induction of ISGs indicate that PM21-NK cells enter an antiviral state in response to IFN-I. While PM21-NK cells treated with media from P/V-mutant-infected cells also enter an antiviral state, it is not clear if this involves the same ISG products as direct IFN treatment. These data highlight the importance of using an IFN-I -inducing virus during oncolytic virus NK cell combination therapy to prevent off-target infection of NK cells. Alternatively, NK cells could be pretreated with IFN-I prior to adoptive transfer to induce an anti-viral state.

### 3.4. IFN-I Treatment of PM21-NK Cells Enhances Their Ability to Kill Lung, Neuroblastoma, and Rhabdomyosarcoma Tumor Cells In Vitro

Given the above results that IFN-I produced by oncolytic virus infection of target tumor cells can protect PM21-NK cells from off-target infection and death and our prior work that IFN-I treatment of lung tumor cells enhances NK cell killing [28], we tested the hypothesis that IFN-I has an additional direct role in increasing the cytotoxic capacity of NK cells. To determine how IFN-I treatment of PM21-NK cells affected the kinetics of NK-cell-mediated killing, an IncuCyte instrument was utilized for real-time analysis of tumor cell death as described in prior work [39]. Using A549 cells which were transduced to stably express a nuclear red fluorescence protein (NucLight Red (NLR)), the IncuCyte instrument can record in real time how NK cells kill target tumor cells, which is evidenced by loss of red fluorescent nuclei, reported as the red object count (ROC). PM21-NK cells were left untreated or treated with increasing concentrations of IFN-I for 18 h. After washing, treated NK cells were then co-cultured with naïve A549 NLR at an effector-to-target (E:T) ratio of 0.625:1. The ROC per well was quantified by the IncuCyte instrument every 2 h, normalized to the ROC at time zero (ROC^t0^), and expressed as a percentage of the initial time zero when NK cell addition occurred (ROC/ROC^t0^(%)).

**Figure 6:**
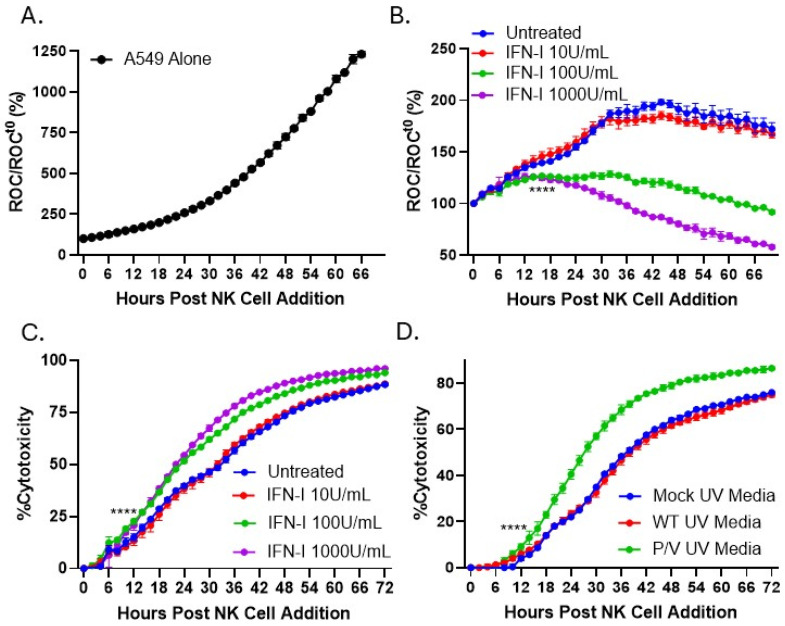
IFN-I or P/V mutant media treatment of PM21-NK cells enhanced NK cell-mediated killing of lung tumor cells. PM21-NK cells were treated with increasing concentrations of IFN-I (0, 10, 100, or 1000 U/mL) (**A**–**C**) or treated with UV-inactivated media from A549 cells that were mock-infected or infected with WT PIV5 or P/V mutant (**D**) and incubated at 37 °C for 18 h. A549 cells expressing a nuclear localized red fluorescent protein were incubated with treated PM21-NK cells. The IncuCyte instrument was used to quantify the red object count (ROC) per well in real time. (**A,B**) The ROC was normalized to the value at time 0 (ROC/ROC^t0^) when NK cells were added and expressed as a percentage. (**C**) The ROC/ROC^t0^ of each well was normalized to the average of the tumor-alone wells, quantifying the percentage of NK cell cytotoxicity towards tumor cells. Values are the mean of three replicates with error bars representing standard deviation. **** indicates when a *p*-value of <0.0001 first appears on the time course, comparing untreated versus 1000 units/mL IFN-I-treated NK cells, and this statistical significance is maintained at later time points.

As shown in Figure 6A, naïve A549 cells alone continue to proliferate with ROC values reaching 1250% of time zero. Figure 6B shows that untreated PM21-NK cells kill A549 tumor cells, causing a gradual decline in the normalized ROC to 180% at 70 h (blue line). Pretreatment of PM21-NK cells with IFN-I causes a concentration-dependent increase in NK-cell-mediated killing of A549 tumor cells, achieving 50% of time zero by 70 h after NK cell addition using the maximum concentration of IFN-I at 1000 units/mL (purple line). Figure 6C shows the same data plotted as the percent cytotoxicity, where PM21-NK-cell killing of A549 cells (Figure 6B) was normalized to the time-matched control wells without NK cells (Figure 6A). These data further highlight the changes in NK cell killing following IFN-I treatment, where IFN-I pretreatment of PM21-NK cells at 1000 units/mL (purple line) results in a 25% increase in cytotoxicity compared to NK cells without pretreatment (blue line).

Culture medium from P/V-mutant-virus-infected tumor cells was tested to determine its ability to also enhance NK cell-mediated killing. A549 cells were mock-infected or infected at an MOI of 10 with WT PIV5 or P/V mutant. Media were harvested and UV treated to inactivate virus particles and used to pretreat PM21-NK cells for 18 h prior to co-culture with naïve A549 NLR cells. As shown in Figure 6D, pretreatment of PM21-NK cells with medium from P/V-mutant-infected A549 cells enhanced NK cell cytotoxicity towards A549 tumor cells (green line) compared to media from mock-infected or WT PIV5 infected cells (blue and red lines).

**Figure 7:**
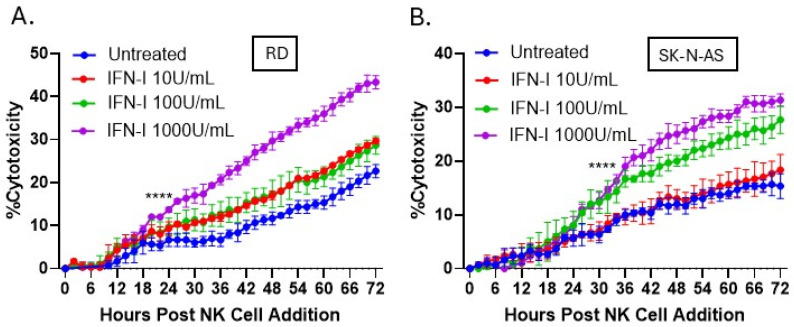
IFN-I treatment of PM21-NK cells enhanced NK cell-mediated killing of pediatric neuroblastoma and rhabdomyosarcoma tumor cells. PM21-NK cells were treated with increasing concentrations of IFN-I (0, 10, 100, or 1000 U/mL) and incubated at 37 °C for 18 h. RD (**A**) or SK-N-AS (**B**) cells expressing a nuclear localized red fluorescent protein were incubated with treated PM21-NK cells. The IncuCyte instrument was used to quantify the red object count (ROC) per well in real time. The ROC was normalized to the value at time 0 (ROC/ROC^t0^) when NK cells were added and expressed as a percentage. The ROC/ROC^t0^ of each well was normalized to the average of the tumor-alone wells, quantifying the percentage of NK cell cytotoxicity towards tumor cells. Values are the mean of three replicates with error bars representing standard deviation. **** indicates when a *p*-value of <0.0001 first appears on the time course, comparing untreated versus 1000 units/mL IFN-I treated NK cells, and this statistical significance is maintained at later time points.

The ability of IFN-I pretreatment of PM21-NK cells to increase cytotoxicity was tested in two additional cell lines: rhabdomyosarcoma (RD cells) and pediatric neuroblastoma (SK-N-AS cells). NLR versions of RD and SK-N-AS cells were co-cultured with PM21-NK cells at an E:T of 0.625 that had been pretreated with increasing concentrations of IFN-I. As shown in Figure 7A,B, IFN-I treatment of PM21-NK cells at 1000 U/mL increased their cytotoxicity nearly two-fold compared to those untreated at 72 h. The overall killing of target cells was slightly lower than seen with A549 target cells as these cell lines are more resistant to NK cell killing, requiring higher E:T for complete killing.

### 3.5. IFN-I or Media from P/V-Mutant-Infected Tumor Cells Upregulates Expression of TRAIL on PM21-NK Cells

**Figure 8:**
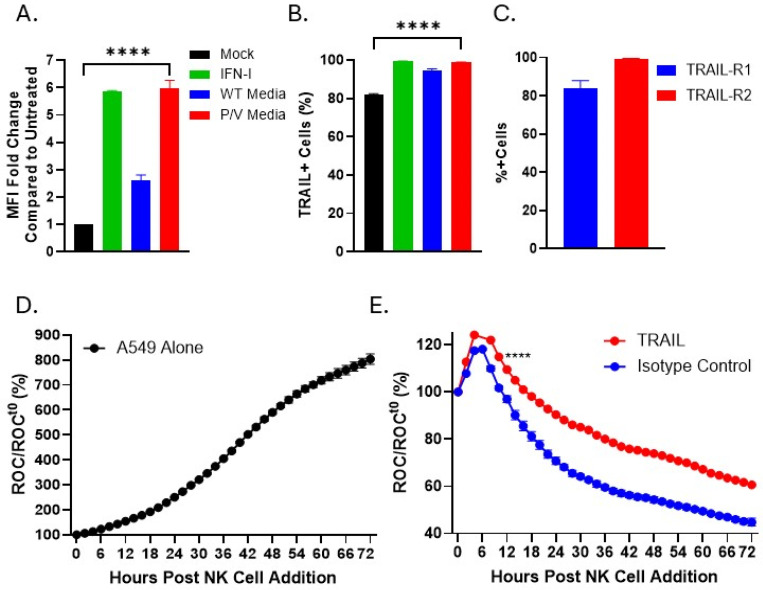
Treatment with IFN-I or UV-inactivated media from tumor cells infected with P/V mutant upregulates TRAIL expression on PM21-NK cells. (**A**,**B**) PM21-NK cells were treated for 18 h with either 1000 U/mL IFN-I or with UV-inactivated media from WT PIV5- or P/V-mutant-infected A549 cells. Cell surface levels of TRAIL was determined by antibody staining and flow cytometry to quantify (**A**) the fold change in mean fluorescence intensity compared to untreated and (**B**) the percent of TRAIL-positive cells in the population. (**C**) A549 cells were stained with antibody for surface expression of TRAIL-R1 and TRAIL-R2. Flow cytometry was used to quantify the percent of positive cells in the population. (**D**,**E**) PM21-NK cells were treated for 18 h with 1000 U/mL IFN-I then incubated with either a TRAIL blocking antibody or isotype control antibodies for 1 h. Antibody-treated NK cells were co-cultured with A549 NLR target cells at an E:T of 5:1. Red object count (ROC) per well was quantified using the IncuCyte and normalized to the ROC at time 0 (ROC/ROC^t0^) when NK cells were added and is expressed as a percentage of time zero. Values are the mean of three replicates with error bars representing standard deviation. **** indicates when a *p*-value of <0.0001 first appears on the time course, comparing TRAIL antibody blocked versus isotype control treated NK cells, and this statistical significance is maintained at later time points.

We examined an NK cell ligand involved in anti-tumor function, tumor necrosis factor-related apoptosis-inducing ligand (TRAIL), which was previously reported to be upregulated with IFN-I [40,41]. Based on the finding that IFN-I upregulates TRAIL expression [42,43], we tested the hypothesis that media from P/V-mutant-infected tumor cells would increase TRAIL expression on the surface of PM21-NK cells. IFN-I treatment of PM21-NK cells induced a six-fold increase in TRAIL surface expression compared to mock-treated cells (Figure 8A). Additionally, treatment with UV media from P/V mutant-infected A549 cells resulted in a six-fold increase in TRAIL expression on the surface of NK cells (Figure 8A). The percentage of NK cells positive for TRAIL increased from 80% in untreated samples to roughly 100% for both IFN-I and P/V UV-media-treated NK cells (Figure 8B). Surface expression of the two TRAIL receptors (TRAIL-R1 and TRAIL-R2) on naïve A549 cells was quantified by flow cytometry. TRAIL-R1 and TRAIL-R2 were present on approximately 80% and 100% of A549 cells, respectively (Figure 8C).

The role of TRAIL in anti-tumor cytotoxicity of PM21-NK cells towards A549 tumor cells was determined by treating PM21-NK cells with IFN-I overnight and then incubating treated PM21-NK cells with anti-TRAIL or isotype control antibodies for 1 h prior to co-culturing with A549-NLR tumor cells. NK cell-mediated killing of A549 NLR was quantified by IncuCyte scanning for ROC every 2 h. As shown in Figure 8D, untreated A549-NLR cells without PM21-NK cells continued to grow, showing the ROC reached a maximum of 800% of time zero. By contrast, PM21-NK cells that received TRAIL blocking antibody (red line) showed reduced ability to kill A549 tumor cells compared to NK cells that received isotype control antibody (blue line, Figure 8E). These data show that treatment of PM21-NK cells with IFN-I or UV media from P/V-infected tumor cells increased TRAIL expression and that TRAIL contributes to maximal PM21-NK-cell-mediated killing of tumor cells in cell culture.

### 3.6. IFN-I and Media from P/V-Mutant-Infected Tumor Cells Decreases IFN-γ Release by PM21-NK Cells

**Figure 9:**
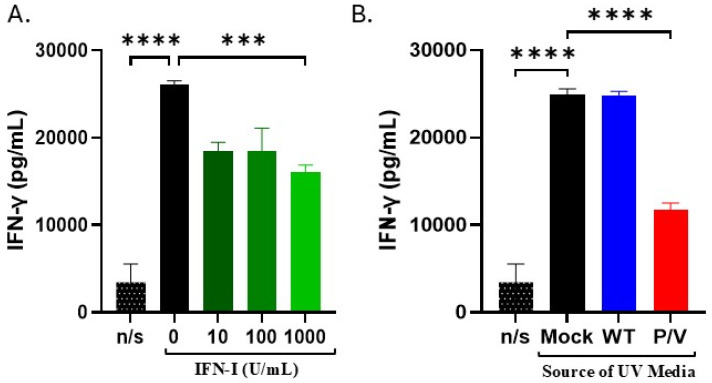
IFN-I or UV media from P/V-infected A549 tumor cells reduced IFN-γ secretion by PM21-NK cells. PM21-NK cells were treated for 18 h with (**A**) increasing concentrations of IFN-I (0, 10, 100, or 1000 units/mL) or (**B**) UV-inactivated media from WT PIV5- or P/V-mutant-infected A549 cells. Cells were left non-stimulated (n/s) or stimulated with an activating cocktail of PMA and Ionomycin for 18 h. Media from treated PM21-NK cells was harvested and analyzed for IFN-γ levels by ELISA. Values in all panels are the mean of three replicates with error bars representing standard deviation. *** and **** indicate a *p*-value of <0.001 and <0.0001, respectively.

Our prior results have shown that when IFN-γ is released from activated PM21-NK cells, it can result in upregulation of NK cell inhibitory ligands on the surface of target tumor cells, leading to reduced NK cell killing [33]. Prior work has shown that IFN-I can negatively alter IFN-γ production by NK cells [44]. Given the above findings, we hypothesized increased cell killing by IFN-I-treated PM21-NK cells could be partly attributed to decreased IFN-γ release and the associated decreased induction of NK inhibitory ligands on tumor cells. As shown in Figure 9A, PM21-NK cells produced high amounts of IFN-gamma after activation, but IFN-gamma levels were decreased in the case of IFN-I treated PM21-NK cells. Additionally, treatment of PM21-NK cells with UV-inactivated media from P/V-mutant-infected A549 cells resulted in decreased IFN-γ release compared to mock or WT UV media (Figure 9B). Together, these data support the hypothesis that IFN-I decreases the release of IFN-gamma from activated PM21-NK cells.

## 4. Discussion

NK cell adoptive immunotherapy is a promising cancer therapeutic modality currently undergoing extensive clinical trial testing [45,46]. Oncolytic viruses can be effective cancer therapeutic agents due to their ability to directly target and lyse tumor cells, induce cytokine signaling that activates the immune system, and modulate expression of ligands on the surface of infected tumor cells [27,28,47,48]. Recently, there has been intense interest in combining oncolytic virus infection of tumor cells with NK cell adoptive immunotherapy, as this combination approach has been shown in some cases to reduce tumor burden to a greater extent compared to individual treatment strategies alone [28,49,50]. However, an unintended consequence of this combination therapy is possibility that progeny oncolytic virus produced by the infected target tumor cell could spread to the adoptively transferred NK cells, resulting in reduced effectiveness of NK cell-mediated killing. Here, we have utilized the PIV5 model system along with in vitro expanded PM21-derived NK cells to demonstrate that a type I IFN-producing oncolytic virus promotes NK cell-mediated killing of tumor cells through three distinct mechanisms—(1) protecting NK cells from off-target infection by tumor-cell-derived virus, (2) promoting inherent NK cell killing activity, and (3) reducing secretion of NK-cell immune-suppressive cytokines.

The selectivity of an oncolytic virus for tumor cells can be accomplished by utilizing an IFN-sensitive virus that is unable to replicate in normal cells but that replicates in tumor cells that have defective IFN pathways [15,16]. IFN-I signaling defects are a common feature of many tumor cells, resulting in increases in tumor survival, accelerated proliferation, and increased angiogenesis [51,52,53]. In our model system, WT PIV5 is not a suitable oncolytic virus candidate, in part due to its broad spectrum cell tropism outside of tumor cells and the role of the V protein in blocking IFN-I synthesis and signaling [20,21,22]. In contrast, cells infected with the PIV5 P/V mutant harboring a defective V protein secrete high levels of IFN-I [24], and our prior work supports a selectivity of the P/V mutant virus for tumor versus normal cells [25,26]. Here, our results from comparing the contribution of WT PIV5 and the oncolytic P/V mutant to PM21-NK cell killing of tumor cells highlights three important roles for IFN-I.

Little is known about the frequency and consequences of off-target oncolytic virus infection of NK cells and how this might alter the efficacy of combination therapy in reducing tumor burden. Spread of progeny virions from oncolytic-virus-infected tumor cells to NK cells could reduce the effectiveness of this therapeutic strategy due to virus-induced cytopathic effects on NK cells. Here, we show that PM21-NK cells are susceptible to infection by both WT and P/V mutant PIV5, and infection with both viruses results in loss of NK cell viability. WT PIV5 is largely non-cytopathic to non-lymphoid-derived (e.g., epithelial) cells in culture [22,23], which contrasts with highly cytopathic P/V mutant infections. Our prior work has shown that these two outcomes of WT and P/V mutant infections of epithelial cells are reversed in the case of primary cultures of human dendritic cells: WT PIV5 is highly cytopathic and P/V mutant virus causes non-cytopathic aborted infections [54]. The outcome of PIV5 infection of NK cells was not previously reported. Thus, different combinations of virus and particular cell types can yield cell death or non-cytopathic infections, and this concept is further highlighted by the current finding that both WT and P/V viruses kill PM21-NK cells in tissue culture. These results support the need to develop approaches to limit NK cell infection from tumor-derived oncolytic viruses.

Our results show that PM21-NK cells respond to IFN-I treatment by increasing expression of ISGs and entering an antiviral state that prevents subsequent PIV5 virus infection. Importantly, media from P/V-virus-infected target tumor cells was also able to protect PM21-NK cells from PIV5 infection, and in co-culture experiments there was limited spread of virus from P/V-mutant-infected tumor cells to PM21-NK cells. Together, these data support the importance of using an IFN-I-inducing oncolytic virus to infect target tumor cells, with the resulting IFN-I production preventing off-target infection of NK cells by tumor-derived oncolytic virus. Some tumor cells are defective in their ability to produce IFN, and therefore, an IFN-I-inducing oncolytic virus would have no effect on preventing off-target virus infection of NK cells. To circumvent a lack of IFN production by some tumor cells, ex vivo expanded NK cells could be pretreated with IFN-I to induce an antiviral state prior to adoptive transfer. IFN-III and tumor necrosis factor-α (TNF-α) are also known inducers of antiviral mechanisms [55,56], and interleukins (IL) such as IL-27, are known to interact with NK cells to induce antiviral properties [57,58,59]. The role of other tumor-derived cytokines, in addition to IFN-I, in preventing off-target oncolytic virus infection of NK cells remains to be investigated.

A second striking finding was that IFN-I treatment of PM21-NK cells in vitro directly increased their cytotoxicity towards a variety of tumor cell types, including lung epithelial carcinoma, neuroblastomas, and rhabdomyosarcomas. Furthermore, UV-inactivated media from P/V infected tumor cells resulted in an increase in NK-cell tumor killing. These results are consistent with other findings that IFN-I is an activator of immune responses and is reported to promote NK cell cytotoxicity [31,60]. While prior work suggests that IFN-I potentiates NK cell cytotoxic activity by increasing perforin-dependent cytotoxicity or maintaining proliferation [31], it is not yet clear if this also applies to PM21-NK cells in the oncolytic virus situation studied here. The direct effects of IFN-I on tumor cells can be determined by the strength and duration of stimulation, with strong and acute IFN-I responses being cytotoxic in tumor cells versus weak and chronic responses that promote tumor cell survival [61]. Therefore, an oncolytic virus that promotes high levels of IFN-I secretion from tumor cells will not only protect NK cells and potentiate their activities but will also provide support for inherent tumor cytotoxicity.

In addition to NK-cell-mediated killing by release of cytotoxic granules, perforin and granzyme, activated NK cells can induce tumor cell death through death receptor pathways [3]. Tumor necrosis factor-related apoptosis-inducing ligand (TRAIL) is a death ligand on NK cells that causes activation of death pathways in tumor cells following interaction with its receptors, TRAIL-R1 and TRAIL-R2 [62]. Consistent with their functions as antiviral immune cells, NK cell cytotoxicity can increase in response to IFN-I through upregulation of TRAIL [43,63]. Supporting these findings, we observed an increase in TRAIL expression on the surface of PM21-NK cells following treatment with purified IFN-I or with UV-inactivated media from P/V infected tumor cells. Further, the functional consequences of TRAIL on NK cells were confirmed as shown by the decrease in PM21-NK-cell tumor killing following antibody blocking of TRAIL. While increased NK cell cytotoxicity in response to IFN-I exposure is likely due to the cumulative effect of various intra- and extracellular changes, our data support IFN-I upregulation of TRAIL as at least one contributing factor.

IFN-γ has been shown to induce upregulation of NK cell inhibitory ligands on the surface of tumor cells, resulting in resistance to NK cell killing [33,64]. Further, while high concentrations of IFN-γ are reported to be anti-tumorigenic, low concentrations can be pro-tumorigenic [65,66]. Therefore, reducing the secretion of IFN-γ from activated NK cells could be beneficial in reducing the resistance of tumor cells to NK cell killing, although it is unclear if it will affect the direct impact on tumor growth. Thus, here we show a third positive consequence of NK cell exposure to IFN-I: PM21-NK cells exposed to IFN-I showed a decrease in IFN-γ secretion, which was also seen following treatment of NK cells with UV-inactivated media from P/V-infected tumor cells. Activation of STAT-1 or STAT-4 in response to IFN-I treatment can result in conflicting gene expression outcomes that can promote or inhibit IFN-γ secretion by NK cells [31,67,68,69]. Early exposure of NK cells to IFN-I causes preferential phosphorylation of STAT-4 resulting in IFN-γ production. In contrast, chronic IFN-I exposure in NK cells leads to inhibition of IFN-γ production due to preferential phosphorylation of STAT-1. The preferential phosphorylation of STAT-1 and STAT-4 in PM21-NK cells in response to IFN-I treatment over time remains to be fully elucidated and will help to further our understanding towards improving NK cell and oncolytic virus combination therapies.

In summary, our results provide new information on the effects that an IFN-I inducing virus can have during oncolytic virus-NK cell combination therapy, outside of the role in onco-selectivity for tumor cells versus normal cells. We provide new information on three mechanisms, whereby IFN-I exposure can enhance NK cell activity against both oncolytic virus infected cells and non-infected cells: (1) generate an antiviral state in NK cells which prevents off-target infection and virus-induced cytopathic effects, (2) increase inherent cell killing capacity of PM21-NK cells through pathways at least in part involving TRAIL, and (3) reduce IFN-γ production.

## Data Availability

The original contributions presented in this study are included in the article. Further inquiries can be directed to the corresponding author.

## Supplementary Materials

The following supporting information can be downloaded at: https://www.mdpi.com/article/10.3390/v17070897/s1, Figure S1: Representative flow cytometry data demonstrating the gating strategy for TRAIL positive NK cells; Figure S2: (A) Representative flow cytometry data demonstrating the gating strategy for NK cells. Dot plots of FSC (area) vs. FSC (height) were gated on single cell population (singlets). From the singlets population, an FSC vs. SSC dot plot was generated and gated on lymphocytes. From the lymphocytes population, a dot plot was generated and gated on CD56 positive NK cells. (B) Gating strategy for percent of live cells using propidium iodide (PI) staining. Mock, WT PIV5, and P/V mutant-infected NK cells at 24 hpi. From the lymphocytes population, a dot plot was generated and gated on PI negative cells.
