# Supplementary material for: A Type I IFN-Inducing Oncolytic Virus Improves NK Cell-Mediated Killing of Tumor Cells In Vitro Through Multiple Mechanisms
Source: Viruses. 2025 Jun 25;17(7):897. doi: 10.3390/v17070897 (PMC12297982; doi:10.3390/v17070897)
Supplement: Supplementary file 1 [file viruses-17-00897-s001.zip › viruses-3716325-supplementary.pdf]

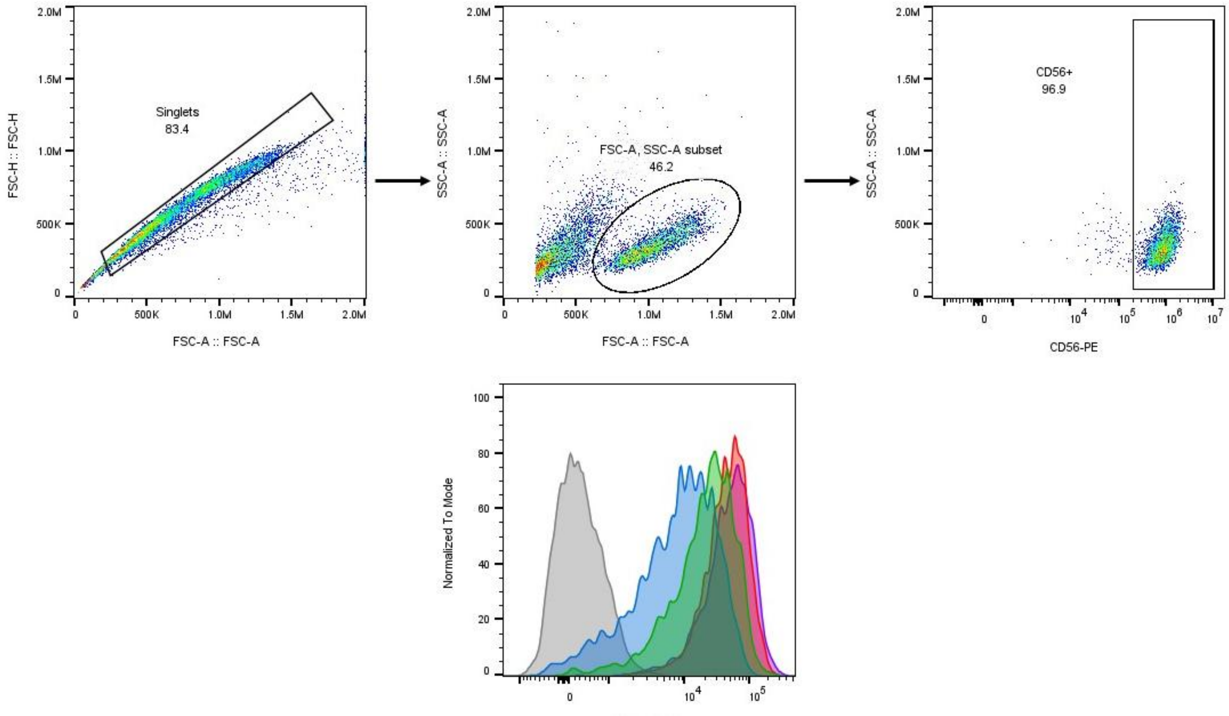

Supplemental Figure S1: Representative flow cytometry data demonstrating the gating strategy for TRAIL positive NK cells. Dot plots of FSC (area) vs. FSC (height) were gated on single cell population (singlets). From the singlets population, an FSC vs. SSC dot plot was generated and gated on lymphocytes. From the lymphocytes population, a dot plot was generated and gated on CD56 positive NK cells. From the CD56 positive NK cell population, TRAIL positive cells were gated.

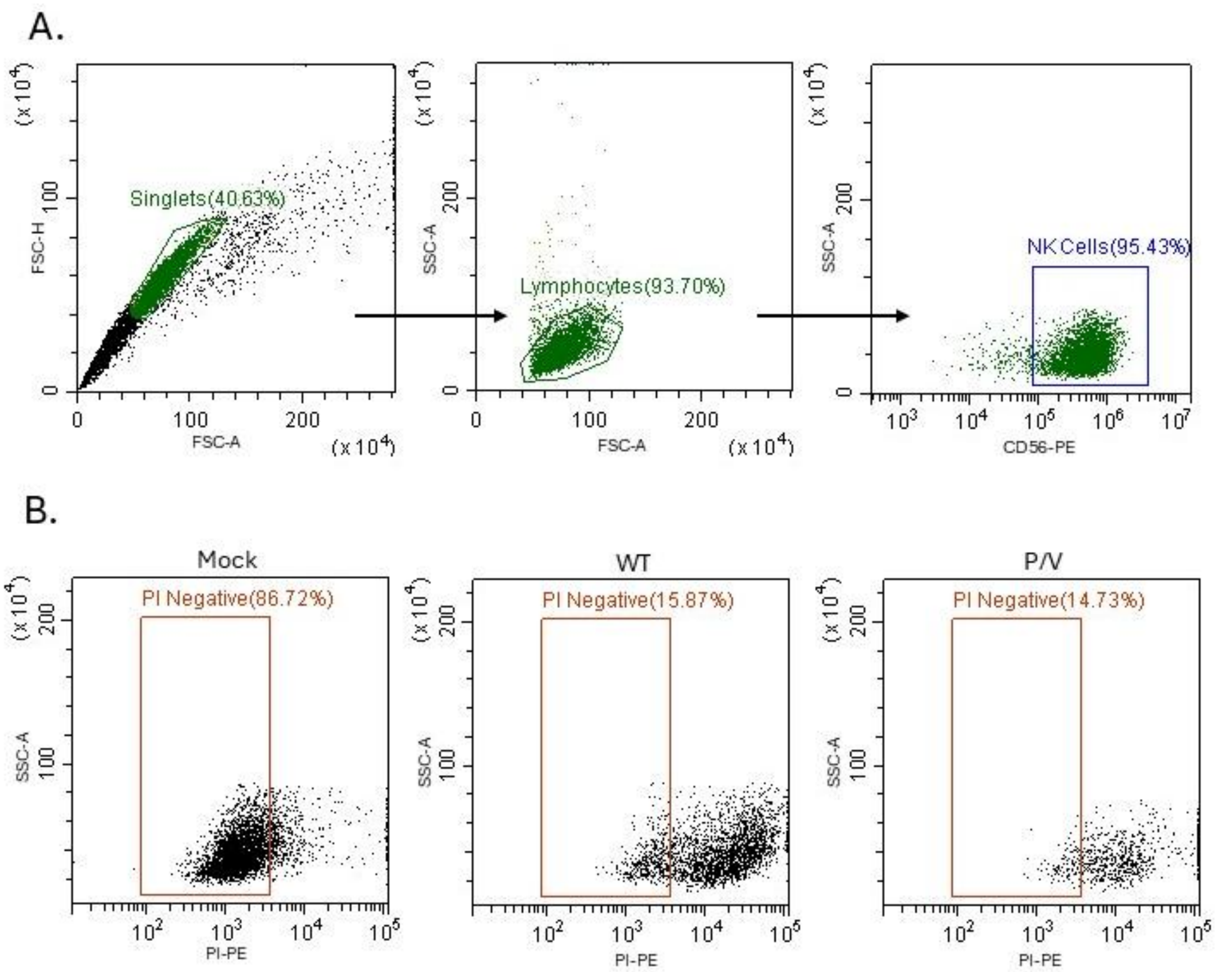

Supplemental Figure S2: A) Representative flow cytometry data demonstrating the gating strategy for NK cells. Dot plots of FSC (area) vs. FSC (height) were gated on single cell population (singlets). From the singlets population, an FSC vs. SSC dot plot was generated and gated on lymphocytes. From the lymphocytes population, a dot plot was generated and gated on CD56 positive NK cells. **B)** Gating strategy for percent of live cells using propidium iodide (PI) staining. Mock, WT PIV5, and P/V mutant-infected NK cells at 24 hpi. From the lymphocytes population, a dot plot was generated and gated on PI negative cells.
